# Influence of Probiotics Feed Supplementation on Hypopharyngeal Glands Morphometric Measurements of Honeybee Workers *Apis mellifera* L.

**DOI:** 10.1007/s12602-023-10107-0

**Published:** 2023-06-19

**Authors:** Ashwak Abdel-Moneim Hassan, Yasser Essam Elenany

**Affiliations:** 1 Department of Dairy Science, Faculty of Agriculture, Cairo University, Giza, Egypt; 2 Department of Economic Entomology and Pesticides, Faculty of Agriculture, Cairo University, Giza, Egypt

**Keywords:** Honeybee workers, Hypopharyngeal glands, Probiotic bacteria, *Lactobacillus brevis*, *Lactobacillus casei*, *Enterococcus faecalis*

## Abstract

More scientific study and methods that are compatible with the honeybee-specific probiotic bacteria are needed in modern beekeeping to increase the productivity and well-being of honeybees. The goal of the current study set out to investigate the possible effects of probiotics previously isolated from the honeybee intestinal tract and soybean patties on nurse worker bee hypopharyngeal gland (HPG) development. The experimentation was carried out in four different treatment groups in which probiotics and soybean patties were provided in different proportions, with control colonies. Results showed that there was a significant increase in HPG morphometric parameters of bees in all experimental groups. Control nurse worker fed with sugar syrup for only 2 weeks had the smallest HPG morphometric parameters. The highest HPG diameter 14.89 ± 0.097 µm and surface area 0.065 ± 0.001µm^2^ were observed in the bees group fed with both probiotic and soya patty. Additionally, the same trend was observed in all morphometric parameters with the bees group fed with probiotic bacteria and soya patty. More royal jelly can be produced by larger HPGs than by smaller ones. Thus, the use of probiotics as a natural alternative tool boosted the development of *Apis mellifera* nurse workers’ HPG that will positively affect the beekeepers’ economy by providing a higher yield of royal jelly production. Overall, the study’s findings show that probiotics are a useful feed supplement for honeybees.

**Supplementary Information:**

The online version contains supplementary material available at 10.1007/s12602-023-10107-0.

## Introduction

Because they pollinate a wide range of crops that are used by both people and animals, honeybees are crucial to agriculture. However, the number of honeybee colonies worldwide has not kept up with the escalating demand [1]. Colony losses have worsened by several interacting biotic and abiotic factors [2, 3]. Nutrition is critical to the ability of an organism to survive difficulties and challenges throughout its lifecycle. Optimal nutrition throughout colony growth, either through access to diverse natural forage [4] or as nutritional supplements [5]. Healthy colony growth results from increased brood production which is dependent on the presence of a healthy population of nurse bees within the colony that produce appropriate brood food via glandular secretions [6]. Glands that produce food provisioned for brood development are the mandibular and hypopharyngeal glands (HPGs) [7, 8]. HPGs are made up of several acini grouped in coils and loops. The largest gland in the skull cavity is thought to be HPG. Each HPG has a thousand or more pear-shaped lobules linked to a long duct, and each lobule is further made up of numerous single-celled glands [9]. The size of HPGs has long been used as a reliable marker of honey bee nutritional status [10]. Additionally, royal action is a key component that facilitates the transformation of growing larvae into queens morphologically [11]. Because queens eat royal jelly, they live longer than normal bees. As a result, the growth of HPGs and the secretory activity of glands directly influence worker behavior, and the colony’s viability may also be impacted. Due to their ability to increase animal output and have growth-stimulating qualities, some phytogenic chemicals received additional attention in the recent past. Previous studies have shown that probiotics improved longevity, productivity, and pathogen tolerance [12, 13]. Here, we determine the HPG size in nurse bees supplemented with probiotic bacteria isolated from the bee’s intestinal tract [14, 15].

“Live microorganisms that, when administered in sufficient proportions, confer a health benefit to the host” is how probiotics are described by ISAPP [16]. Originally, probiotics were implemented to promote immunological function, lower blood cholesterol, and prevent cancer by enhancing gut health, immune response, and both animal and human health [17]. Among the known probiotic microorganisms, species of lactic acid bacteria (LAB) (such as *Lactococcus*, *Lactobacillus*, *Streptococcus*, and *Enterococcus*) and *Bifidobacterium* have a long history of safe use [18]. Most of the research on the microbes connected to honeybees has been done with the aim of identifying and isolating the helpful bacteria connected to these bees. While substantial research has been done on the impact of stingless bee honey and probiotic LAB isolated from honeybees on honeybee pathogens, little is known about the physiological effects of probiotic bacteria in addition to how they affect colony reproduction. It is worth noting that there has not been much research on how probiotic feed affects the morphometric characteristics of the hypopharyngeal gland. Therefore, the aim of this study was to investigate the effect of probiotic bacteria as an additive to the supplementary feed on the size of nurse worker bees’ HPGs and the amount of secreted royal jelly.

## Materials and Methods

### Ethical Statement

The current research did not need any specific permits. Nurse worker bees for the experiments were obtained from experimental apiary of Cairo University’s Faculty of agriculture, Giza, Egypt. *Apis mellifera* in Honeybee Research Garden is not a protected or endangered species.

### Experimental Honeybee Colonies Setup

According to the procedure outlined by William et al. [19], workers of *A. mellifera* aged 0 to 24 h were gathered by putting the frames containing a large amount of sealed brood in an incubator for 24 h at 30 °C temperatures and 70% humidity. The newly emerging workers were then moved into plastic cages with the dimensions 11 cm × 6 cm × 6 cm [9]. In an incubator, a bowl with a steel mesh top was also added to regulate humidity and prevent the newly emerged bees from falling into the bowl. In a plastic syringe feeder with a 5-mL capacity and a 3-mm pore, the sugar syrup was given to caged bees. Similarly, soybean patties were prepared using the technique outlined by [9]. The cages containing the fifty newly emerged bees were transferred right away to an incubator that was kept at 30 °C and 70% humidity [20, 21]. In 2022, an experiment was conducted from March to June.

### Probiotics Culturing, Dose Preparation, and Counting C.F.U.

Tables 1 and 2 summarize the characteristics and administration information of the probiotic bacterial species employed in the current study. All strains used were isolated from the honeybee intestinal tract and molecularly identified based on the 16S rRNA gene sequences [14, 15].

**Table 1:** Name and source of probiotics administered to the experimental bees

Probiotics	Source of isolation	Probiotics (C.F.U./mL of sugar syrup)
*Enterococcus faecalis*-HBE1	Isolated from honeybee rectum	1** × **10^7^
*Lactobacillus brevis*-HBE2	Isolated from honeybee rectum	
*Enterococcus faecalis*-HBE3	Isolated from honeybee rectum	
*Enterococcus faecalis*-HBE4	Isolated from honeybee mid gut	
*Lactobacillus casei*-HBE5	Isolated from honeybee stomach

**Table 2:** Detail of probiotics and soya bean patty administration to honeybees in different experimental groups

Experiment	Experimental code	Detail
Control (C)	––––––––––––	Sucrose in DW (1: 1 V/V%)
Treatment (1)	Ex_1_	Soya bean patty (soya bean flour 50% sugar syrup 50%)
Treatment (2)	Ex_2_	*Enterococcus faecalis*-HBE1, HBE2, and HBE3. *Lactobacillus casei*-HBE5 and *Lactobacillus brevis*-HBE2 in sugar syrup
Treatment (3)	Ex_3_	Soya bean patty 50% probiotic strains 50%
Treatment (4)	Ex_4_	Soya bean patty 30% probiotic strains 70%

Probiotic lactic acid bacterial strains were cultivated in MRS medium at 37 °C in a 5% carbon dioxide prior to incubation for overnight (18 h). The inoculum count was adjusted at OD_600_ = 0.1 (10^7^ CFU/mL) using a spectrophotometer. Subsequently, 1 mL of the MRS broth with 10^7^C.F.U. and 0.1 O.D. was taken in an Eppendorf tube and it was centrifuged at 15,000 rpm for 10 min at 25–30 °C. Later on, the supernatant was discarded, and the pellet was maintained in 15% glycerol and stored at − 20 °C. According to Martin-Hernandez et al. [22], sugar syrup was made by mixing sucrose and distilled water (1:2). With light modifications of C.F.U. and vehicle in the experiment of Hassan et al. [9], in the current experiments, 1 × 10^7^C.F.U. and sugar syrup were provided with probiotic bacteria pellets and the bees were fed on soya bean patty. Respective sugar syrups in 100-mL quantity and soya bean patty 100 g were provided to cage twice a week.

### Nurse Worker Samples and Heads Dissection

Samples of nursing bees at different ages, ranging from 1 to 15 days, were collected at the time of introduction into the colonies and then every 5 days after that. Five workers from each age and experimental group were sampled. The heads of the studied worker bees were fixed using two entomological needles on a rubber base (Xantopren^®^ L blue and Activator universal, Heraeus Kulzer, Germany) in Hyes’ solution (NaCl 9.0 g, KCl 0.2 g, CaCl 0.2 g, NaHCO3 0.1 g, 1 l distilled water, pH 8.5) on a petri dish. For dissection, the stereomicroscope was employed (SterREO Discovery.V12, Zeiss). The external chitinous exoskeleton of the head’s facial region was removed between the compound eyes in order to collect the proper morphometric measurements of the glands.

### Hypopharyngeal Glands Morphometric Parameters Using Image Analysis

The hypopharyngeal glands were photographed using a scanning electron microscope (SEM). To fix the protein, the HPG samples were fixed for 2 h in 3% glutaraldehyde in 0.1 M phosphate buffer at pH 7.2. In 15-min intervals, samples were washed numerous times in 0.1 M phosphate buffer (pH 7.2). They were then dehydrated for 5 min each in a series of aqueous ethanol solutions (25%, 50%, 75%, 95%, and 100%). The samples were placed on aluminum SEM stubs and sputter-coated with gold after being dried to critical point with CO_2_ in a critical point dryer (Polaron, Waterford, England) (SPI module sputter coater, SPI Supplies Division of Structure Probe, Inc). Samples were analyzed using a scanning electron microscope at 10 to 25 kV [14]. For image analysis, Scanning Prop Image Processor (SPIP) program 8 (BETA, Denmark) was used which enables the user to manipulate lateral calibration and unit cell detection to account for magnification differences in each image.

After calibration of the program according to the scale bar on the micrographs, HPG images were magnified for better definition. Detection and quantification of HPGs were done using the polygon measure shape. The program was keeping the measurements in memory and calculating some statistical values. Several morphological and geometrical parameters such as diameter, area, length, breadth, perimeter, and roundness were calculated by the system.

### Measuring of Royal Jelly Yield

According to the method described by Khan and Ghramh [23], a microspatula was used to collect RJ from the cells into a plastic container, and its weight was calculated with an electronic scale (AL204-IC, Mettler Toledo, Switzerland). RJ was collected and stored at − 20 °C in the fridge.

### Statistical Analysis

Mean ± standard deviation (SD) values are used to express the data. A randomize complete block design and analysis of variance of factorial methods were done using Mstat 4.0 statistical software (Norman Drinkwater, McArdle Laboratory). All data were analyzed in three replications for each parameter. The least significant differences (LSD) test was applied to compare the significant differences between the mean values for different treatments. At *p* < 0.05, the results were found to be statistically significant.

## Results

**Fig. 1:**
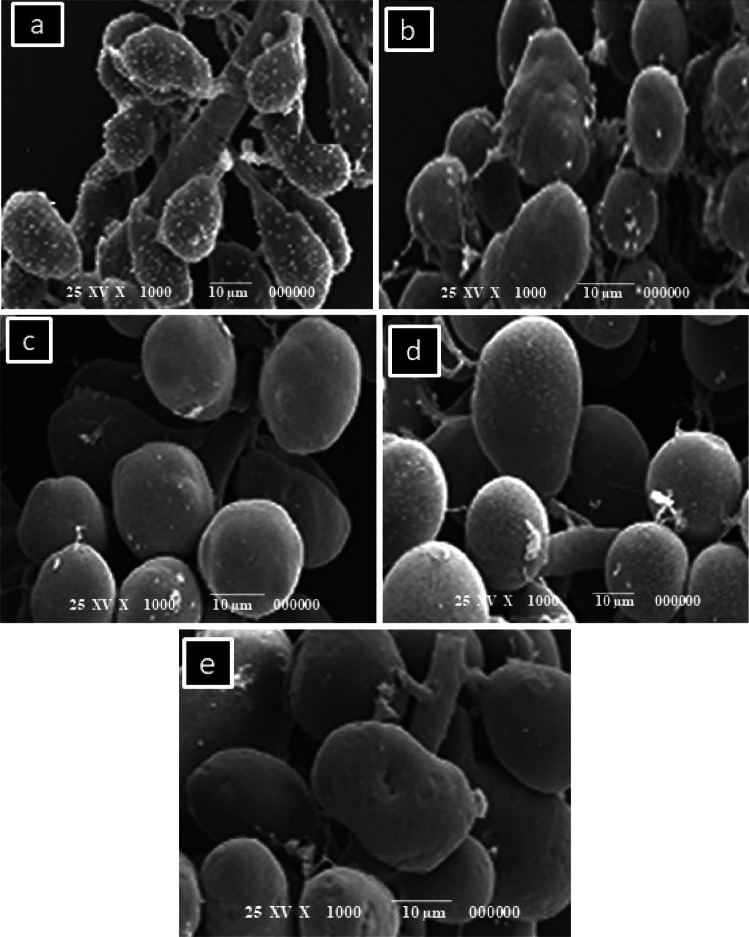
Scanning electron micrographs show hypopharyngeal gland (HPG) morphology of bees fed with sugar syrup as a control (**a**), soya bean patty (**b**), probiotic bacteria (**c**), soya bean patty 50% probiotic strains 50% (1:1) (**d**), and probiotic strains 70% soya bean patty 30% (2:1) (**e**)

Results showed a favorable effect of probiotic feed supplementation on the development of HPGs. The glands were photographed, and representative images are shown in Fig. 1. Commonly, different scales are used in vertical and horizontal axes. It is typical, when using SEM, to observe a 3-dimensional spherical object such as glands that the central region of the image is darker than the periphery. SEM observations revealed that the HPG took the shape of long clusters surrounding an elongated central axial duct. The glands were paired structures composed of numerous secretory units, or acini that were connected to the central axial duct by thin, individual, excretory canaliculi. As shown in Fig. 1, larger acini size has come from feeding the bees with probiotic bacteria and soya patty as a source for protein compared to the control group which fed on sugar syrup. Because the hypopharyngeal gland size is sensitive to the amount of protein and pollen in the diet and is a critical marker of nourishment in bees, the greatest acinal area of HGs was observed when honeybees fed on probiotic bacteria and soya patty mixture (2:1) followed by probiotic bacteria and soya patty mixture (1:1), while honeybees fed on sucrose solution had a lower size of HGs compared to those fed on probiotic bacteria.

Control bees which were fed with sugar syrup for 2 weeks depicted the mean value of 13.07 ± 0.158 µm for diameter while bees of treatment (4) were fed with probiotic bacteria and soya bean patty recorded 14.89 ± 0.097 µm for diameter. Likewise, worker bees of treatment (1) and treatment (4) showed mean values of surface area as 0.047 ± 0.001 and 0.065 ± 0.001 µm^2^, respectively. Statistically significant differences (LSD = 0.1458, 0.1031) were found within HPG diameters and surface area means as a function of feeding formula (*α* = 0.05). The feeding treatments affected the HPG diameter and surface area significantly (*P* < 0.001) as shown in Table 3. Roundness was measured through the two-dimension image analysis. The maximum roundness for the tested HPGs was 0.604 ± 0.60 for treatment (4) and the minimum was 0.431 ± 0.06 for the control.

Roundness of HPGs showed more samples far away from being a perfect circle as found for treatment (2) and treatment (3) (0.523 ± 0.15, 0.548 ± 0.25), respectively, which indicated irregular circles (Fig. 1). It was worth mentioning that it was impossible to measure the length of many HPGs due to their curved shape. Additional1y, the glands’ edges were, in many cases, difficult to measure. For that gland, images with a straight and clear visibility were measured. The maximum and minimum length values of are presented in Table 3. Great differences (*P* < 0.001) were found in the length of the hypopharyngeal glands. In addition, wide perimeter ranges from 4.02 μm for the control to 4.74 μm for bees fed with probiotic bacteria and soya bean patty. Significant influence (*P* < 0.05) for perimeters was found as a function of type of feeding (LSD = 0.5834 at *α* = 0.05). By comparing data of the hypopharyngeal gland image, it becomes evident that there is a significant difference within width of the glands.

**Table 3:** Average hypopharyngeal glands diameter, length, breadth, perimeter (µm), and surface area µm^2^

**Treatments**	**Diameter** µ**m**	**Surface area** µ**m**^**2**^	**Length** µ**m**	**Breadth** µ**m**	**Perimeter**	**Roundness**
Control (C)	13.07 ± 0.158^e^	0.047 ± 0.001^e^	57.26 ± 0.158^e^	42.73 ± 0.111^e^	4.021 ± 0.152^e^	0.431 ± 0.06^e^
Ex _(1)_	13.79 ± 0.207^d^	0.050 ± 0.001^d^	59.32 ± 0.306^d^	43.66 ± 0.214^d^	4.072 ± 0.111^d^	0.483 ± 0.26^d^
Ex _(2)_	14.03 ± 0.301^c^	0.059 ± 0.002^c^	64.52 ± 0.168^c^	44.93 ± 0.461^c^	4.148 ± 0.312^c^	0.523 ± 0.15^c^
Ex _(3)_	14.69 ± 0.108^b^	0.061 ± 0.002^b^	70.60 ± 0.220^b^	46.80 ± 0.300^b^	4.324 ± 0.203^b^	0.548 ± 0.25^b^
Ex _(4)_	14.89 ± 0.097^a^	0.065 ± 0.001^a^	72.13 ± 0.289^a^	47.06 ± 0.221^a^	4.748 ± 0.318^a^	0.604 ± 0.60^a^

Data means ± SD (n = 3). Different superscript letters in the same column indicate significant difference (*P* < 0.001)

**Fig. 2:**
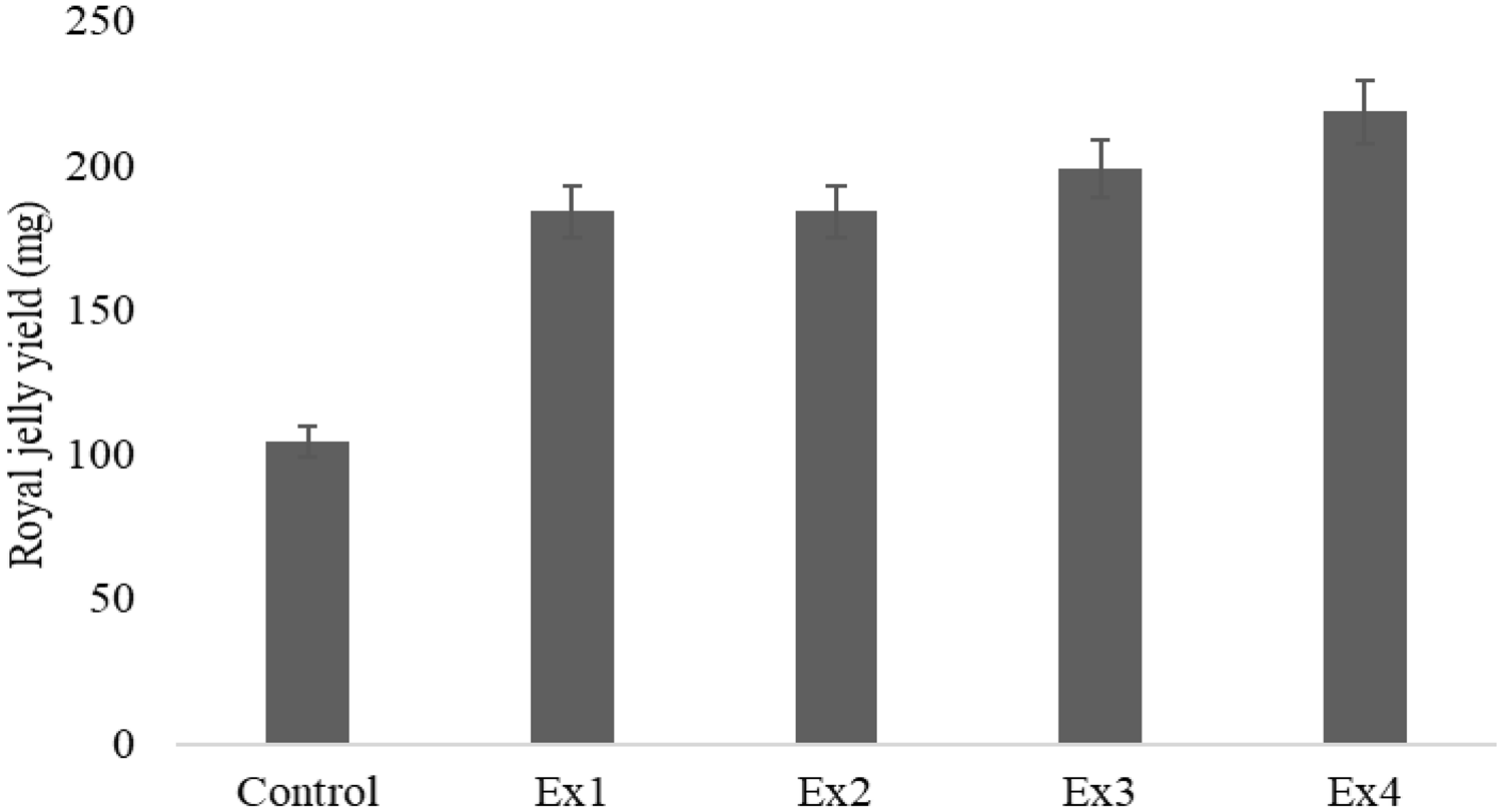
Royal jelly yield per cell cup (mg) for control, Ex_1_ soya bean patty, Ex_2_ probiotic bacteria, Ex_3_ (soya bean patty 50% and probiotic 50% (1:1)), and Ex_4_ probiotic 70% and soya bean patty 30% (2:1)

The average weight of royal jelly (RJ) per cup (mg) between different treatments compared to the control is mentioned, respectively (Fig. 2). The RJ yield was statistically greater in bees fed on probiotic bacteria mixed with soya patty EX_3_ and Ex_4_, respectively, in comparison to control bee colonies (*p* < 0.001). The maximum RJ yield was 219.23 ± 0.51 mg in bees fed with probiotic bacteria and soya patty (2:1) (Ex_4_). Similarly for bees fed on probiotic bacteria 50% and soya patty 50% (Ex_3_), the RJ yield was 199.273 ± 0.76 mg, while in the bees fed with soya patty the RJ yield was 184.57 ± 0.33 mg and the bees fed on probiotic bacteria the RJ yield was 184.11 ± 0.23 mg. The amount of RJ yield does not show statistically significant differences among both bees fed either probiotic bacteria only or soya bean patty diet.

## Discussion

Understanding the morphogenesis of honeybee HPGs and the factors which regulate HPG development is essential for further investigations of the functionality of the glands [24]. HPGs are age-dependent structures in honeybees that change with the acinus size and correlate with different social behavior, especially, the quantity and quality of food as mentioned by [25, 26]. Nurse hypopharyngeal glands produce the protein fraction of the worker and royal jelly that is fed to developing larvae and queens [27]. The glands get smaller when nurses are fed deficient diets and are large when they are fed complete diets. Mao et al. [27] mentioned that nurse hypopharyngeal gland size is a robust indicator of nurse nutrition and health which can be improved with the use of probiotics. We noticed an increase in HPG size and in bees that were fed with probiotics. The results can be explained by the fact that the physiological status of bees reacted positively with the presence of beneficial microbes in the food as reported by [28, 29]. Feeding bee colonies on sugar syrup incorporating soya bean led to the development of glands greater in diameter than those of bees in the control group, likewise, in surface area, perimeter, length, and roundness. Bees given probiotic supplement in their feed developed hypopharyngeal gland greater in morphometric parameters than those in the control group, while supplementation of feed with both soya bean patty as a source for protein and a probiotic bacteria led to a better development of glands than those found in bees fed pure sugar syrup or soya patty. Mentioned results of HPG morphometric parameter agreed with some authors as [9, 30–32]. This greater development of glands as a result of feeding bees with probiotic is correlated with increased gland size resulting in increased royal jelly production. Our results agree with those of [33–35].

Royal jelly yield increased by increasing the HPG size resulting from probiotic feed. This greater development of HPG cells as a result of feeding bees with probiotic and soya bean patty is correlated with increased glandular activity resulting in increased RG production. However, more research is required to better understand how different probiotic strains affect the RJ yields.

## Conclusions

It is worth noting that incorporation of probiotics in honeybee’s feed proves good for pollinator’s overall health. This study helps to obtain higher royal jelly yields. Future studies on the assessment of various other probiotic blends at pilot and commercial scales are needed for obtaining exact quantitative results at higher scales.

### Supplementary Information

Below is the link to the electronic supplementary material.

Supplementary file1 (DOCX 1343 kb)

## Data Availability

The datasets generated during and/or analyzed during the current study are available from the corresponding author on reasonable request.
