# Supplementary material for: Influence of Probiotics Feed Supplementation on Hypopharyngeal Glands Morphometric Measurements of Honeybee Workers Apis mellifera L
Source: Probiotics Antimicrob Proteins. 2023 Jun 19;16(4):1214–20. doi: 10.1007/s12602-023-10107-0 (PMC11322241; doi:10.1007/s12602-023-10107-0)
Supplement: Supplementary file 1 — Supplementary file1 (DOCX 1343 kb) [file 12602_2023_10107_MOESM1_ESM.docx]

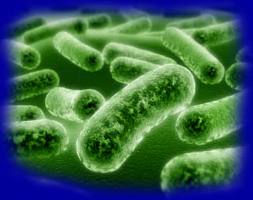

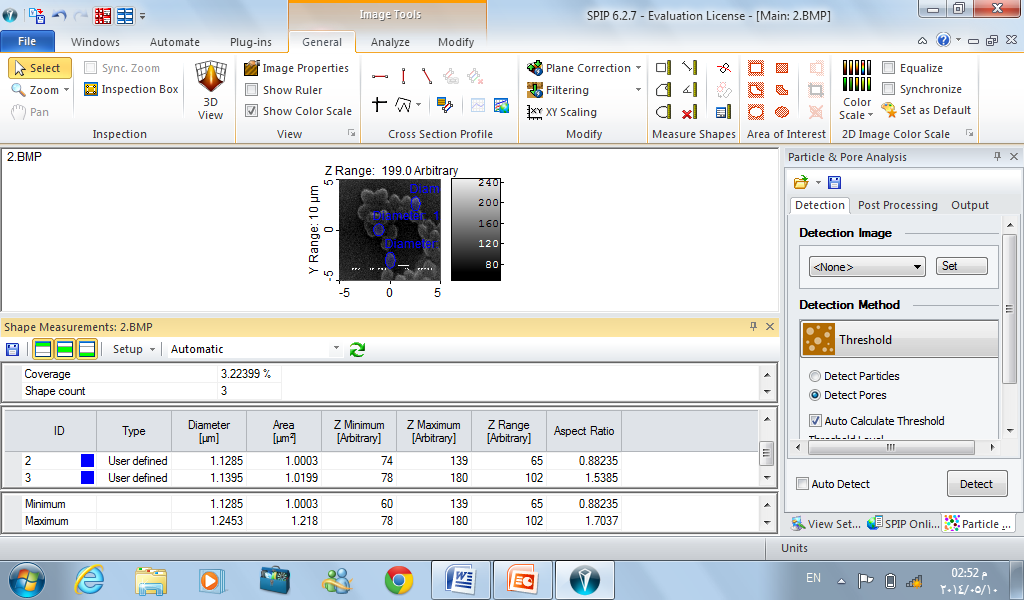

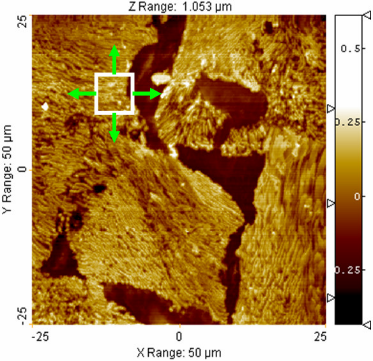


Geometrical properties of glands

Shape and Orientation Parameters


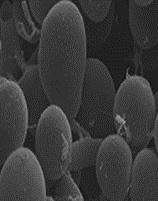


**25 XV X 1000 10 µm 000000**

**Supplementary File 1: Screen shot of measurement SPIP software while measuring the gland morphometric measurements and creating the report**
